# Avian *Plasmodium* in Eastern Austrian mosquitoes

**DOI:** 10.1186/s12936-017-2035-1

**Published:** 2017-09-29

**Authors:** Ellen Schoener, Sarah Susanne Uebleis, Julia Butter, Michaela Nawratil, Claudia Cuk, Eva Flechl, Michael Kothmayer, Adelheid G. Obwaller, Thomas Zechmeister, Franz Rubel, Karin Lebl, Carina Zittra, Hans-Peter Fuehrer

**Affiliations:** 1 Department of Pathobiology, Institute of Parasitology, University of Veterinary Medicine Vienna, Veterinaerplatz 1, 1210 Vienna, Austria; 2 Division of Science, Research and Development, Federal Ministry of Defence and Sports, Vienna, Austria; 3 Biological Station Lake Neusiedl, Burgenland, Austria; 4 Department for Farm Animals and Veterinary Public Health, Institute for Veterinary Public Health, University of Veterinary Medicine Vienna, Veterinaerplatz 1, 1210 Vienna, Austria

## Abstract

**Background:**

Insect vectors, namely mosquitoes (Diptera: Culicidae), are compulsory for malaria parasites (*Plasmodium* spp.) to complete their life cycle. Despite this, little is known about vector competence of different mosquito species for the transmission of avian malaria parasites.

**Methods:**

In this study, nested PCR was used to determine *Plasmodium* spp. occurrence in pools of whole individuals, as well as the diversity of mitochondrial cytochrome b gene sequences in wild-caught mosquitoes sampled across Eastern Austria in 2013–2015.

**Results:**

A total of 45,749 mosquitoes in 2628 pools were collected, of which 169 pools (6.43%) comprising 9 mosquito species were positive for avian *Plasmodium*, with the majority of positives in mosquitoes of *Culex pipiens* s.l./*Culex torrentium*. Six different avian *Plasmodium* lineages were found, the most common were *Plasmodium vaughani* SYAT05, *Plasmodium* sp. Linn1 and *Plasmodium relictum* SGS1. In 2014, mosquitoes of the *Culex pipiens* complex were genetically identified and *Culex pipiens* f. *pipiens* presented with the highest number of avian *Plasmodium* positives (n = 37; 16.74%). Despite this, the minimum infection rate (MIR) was highest in *Culex torrentium* (5.36%) and *Culex pipiens* f. *pipiens/*f. *molestus* hybrids (5.26%). During 2014 and 2015, seasonal and annual changes in *Plasmodium* lineage distribution were also observed. In both years *P. vaughani* SYAT05 dominated at the beginning of the sampling period to be replaced later in the year by *P. relictum* SGS1 (2014) and *Plasmodium* sp. Linn1 (2015).

**Conclusions:**

This is the first large-scale study of avian *Plasmodium* parasites in Austrian mosquitoes. These results are of special interest, because molecular identification of the taxa of the *Cx. pipiens* complex and *Cx. torrentium* enabled the determination of *Plasmodium* prevalence in the different mosquito taxa and hybrids of this complex. Since pools of whole insects were used, it is not possible to assert any vector competence in any of the examined mosquitoes, but the results are nonetheless valuable in providing an overview of avian *Plasmodium* species and lineages present in Austria.

## Background

Haemosporidian parasites of the genus *Plasmodium* are responsible for avian malaria worldwide. Of the accepted 38 valid species of *Plasmodium* spp. [1] 488 recognised *cytochrome b* lineages are currently described [2]. The richest lineage diversity of *Plasmodium* parasites is present in South America, and in general, the diversity is much higher in tropical ‘hotspot’ areas (e.g., India, Australia, Southeast Asia) than in temperate regions [2]. Four very common avian *Plasmodium* lineages found in birds and mosquitoes in Europe are *Plasmodium relictum* SGS1*, Plasmodium elongatum* GRW6, *Plasmodium vaughani* SYAT05 and *Plasmodium* sp. Linn1 [3–6], with *P. relictum* SGS1 being the most common [7, 8]. These lineages have also been found in Austria in a previous study in birds [9].

Avian *Plasmodium* parasites rely on arthropod vectors to complete their life cycle. For most species, the vectors are mosquitoes of the genera *Culex*, *Aedes, Ochlerotatus*, *Culiseta* and possibly *Anopheles* [1]. *Plasmodium* parasites possess a sexual and an asexual part to their life cycle; the gametogony, fertilization, formation of zygotes, and the sporogony occur in the vector, while merogony and gametocytogony happen in the vertebrate host. If the vector is fully competent, *Plasmodium* parasites reach and fully develop in the salivary glands, from where they are transmitted to the vertebrate host during a blood meal. The vector competence for transmitting these parasites varies between mosquito species, and each *Plasmodium* species may use a number of different mosquito species as vectors [10]. Not all vectors are equally susceptible to avian *Plasmodium.* For example, in a study comparing three vector species on Hawaii, the parasite prevalence between species varied significantly, with *Culex quinquefasciatus* being the most susceptible [11]. To date, a specific list of vectors for *Plasmodium* spp. has not yet been compiled [1, 12] although a list of potential vector species can be found on the MalAvi database [13]. So far, the genus *Culex* seems to provide the most successful vectors worldwide; in different studies it has been found that mosquitoes of this genus contained the biggest diversity of different *Plasmodium* strains [10, 12].

Forty-three indigenous species of the family Culicidae from 8 genera (*Aedes*, *Anopheles*, *Culex, Coquillettidia*, *Culiseta, Ochlerotatus, Orthopodomyia*, and *Uranotaenia*) have been specified in Austria [14, 15]. The establishment of the potential invasive species *Aedes albopictus* (Asian tiger mosquito) in Austria is still in dispute [14]. In addition, 4 more non-native species, *Ochlerotatus japonicus japonicus*, *Anopheles hyrcanus*, *Orthopodomyia pulcripalpis*, and *Culiseta longiareolata* have been reported [14, 15]. The taxon *Culex pipiens* belongs to a complex and is seen in Central and Western Europe in two forms, *molestus* and *pipiens*, which frequently hybridize and cannot be distinguished morphologically [16]. Another species, *Culex torrentium*, while not part of this species complex, is also very difficult to distinguish morphologically [16]. All these species should therefore only be listed as individual taxa apart from the *Culex pipiens* species complex if they have been identified genetically.

Not all of these mosquito species are competent vectors for avian *Plasmodium* lineages. So far, avian *Plasmodium* parasites have been found in the Central European species *Aedes vexans*, *Cx. pipiens* complex, *Cx. modestus*, *Cx. hortensis*, *Culiseta annulata*, *Ochlerotatus caspius* and the alien species *Ae. albopictus* [3, 17–21]. While *Ae. albopictus* has dramatically expanded its distribution range, this mosquito is clearly anthropophilic and has been shown to have a lower prevalence of avian malaria parasites than native *Culex* species [19]. *Culex pipiens* f. *molestus* has been proven in experimental infections to be a competent vector for the *P. relictum* lineages pSGS1 and pGRW11 and GRW4 [22, 23]. While examinations of *Culex pipiens* f. *pipiens* indicate a vector role [24], they are not distinguishable from *Cx. pipiens* f. *molestus*. Knowledge about vector competence for most other mosquito species is currently lacking.

This is the first time that Austrian mosquitoes have been examined for avian *Plasmodium* on a large scale. Thousands of female mosquitoes were screened, collected over three years in three Eastern Austrian provinces, namely Vienna, Lower Austria and Burgenland for avian *Plasmodium*, to gain an overview which parasites are present in the area, as well as diversity and prevalence in different mosquito species. For 2014, the taxa of the *Cx. pipiens* complex/*Cx. torrentium* were genetically identified within another project [25], which allowed to also determine avian *Plasmodium* incidence in morphologically similar *Culex* taxa in more detail.

## Methods

Avian *Plasmodium* DNA for this study was obtained from mosquitoes sampled in two independent monitoring efforts, which also used two different storing conditions (dry and − 80 °C), conducted from 2013 to 2015.

### Mosquito sampling effort 1

In 2013 and 2014, adult female mosquitoes were collected by utilizing new standard miniature light traps (John W Hock Company, Gainesville, FL, USA). These traps were baited with bottled carbon dioxide (Air Liquide, Schwechat, Austria) on a daily basis for 24 h from March to October at three locations in Vienna. Mosquitoes were killed using the insecticide dichlorvos upon entering the trap. Once a week the traps were emptied and mosquitoes were dried and stored at room temperature until further processing.

### Mosquito sampling effort 2

**Fig. 1:**
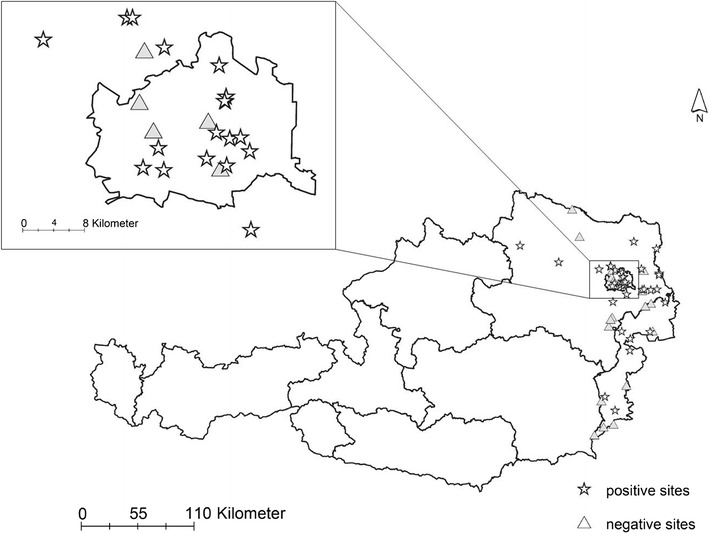
Sampling sites for mosquitoes in Eastern Austria during the years 2013–2015. The close-up provides an overview of the city of Vienna where sampling sites were densest. Sites positive for avian *Plasmodium* are marked by stars, negative sites are marked by triangles

Mosquitoes were monitored across three provinces of Eastern Austria (Burgenland, Lower Austria, Vienna) at 35 permanent and 23 non-permanent trapping sites (Fig. 1). At permanent sampling sites, mosquitoes were collected for a 24-h period on a regular basis every 2nd week from April to October 2014–2015, using Biogents Sentinel Traps (Regensburg, Germany) equipped with bottled carbon dioxide (Air Liquide, Schwechat, Austria) as attractant. Non-permanent sampling sites were sampled at least once and up to six times during the summer months using Biogents Sentinel Traps (Regensburg, Germany) or aspirators. All mosquitoes were stored at − 80 °C until further procedure.

Morphological identification of mosquito species was performed using the identification key of Becker et al. [16]. Mosquito individuals were pooled by species, collection site and date, with a maximum number of 50 individuals (average pool size 17.9, min = 1, max = 50). To identify *Cx. pipiens* taxa genetically, three legs of each individual were taken and processed individually. To each sample, 400 µl of DNA/RNA lysis buffer (Zymo Research Corp., USA) and two ceramic beads (Precellys Ceramic Beads, Peqlab Biotechnologie GmbH) were added and homogenized in a TissueLyser II (Qiagen, Germany). Approximately 350 µl of the homogenized material was loaded onto a QIAshredder (Qiagen, Germany). To filter the samples, the filled QIAshredders were centrifuged for 2 min at 13,000 rpm (solid components of the samples remained on the column). DNA was extracted using a ZR-Duet™ DNA/RNA Miniprep Kit (Zymo Research Corp, USA) according to the manufacturer’s instructions.

For amplifying *Plasmodium* spp. DNA, each DNA sample was then subjected to nested PCR, described by [26] without modification, with the used primers targeting a 480 bp fragment of the mitochondrial cytochrome b gene. PCR products were separated by electrophoresis in 2% agarose gels stained with Midori Green Advance DNA stain (Nippon Genetics Europe, Germany). Finally, purified PCR products were commercially sequenced at LGC Genomics GmbH, Germany. Obtained sequences were viewed and aligned using the programme Genious version 10.0.6 [27]. Then the sequences were compared for similarity to sequences available on the MalAvi [13] and the GenBank^®^ [28] databases.

### Minimum infection rate

To evaluate the infection rate of the collected mosquitoes, the minimum infection rate (MIR) of each mosquito species was calculated. If a mosquito pool was positive for *Plasmodium* on PCR, it was assumed that the pool contained at least one positive individual. Therefore, MIR (percentage) was calculated as follows:

MIR (%) = n_(PCR positive pools)_/n_(total analysed mosquitoes)_ × 100 [5, 29].

## Results

A total of 45,749 mosquitoes of 23 different species were collected in Vienna and Eastern Austria (Fig. 1) in 2013–2015. From these, 2628 pools were created using up to 50 whole mosquito individuals separated by species, time and site of sampling. A total of 169 (6.43%) mosquito pools from 9 identified mosquito species and several pools of unidentified individuals of the genera *Aedes/Ochlerotatus*, *Culex* and *Anopheles* were positive for avian *Plasmodium*. Pools positive for *Plasmodium* were of the following mosquito species: *Ae. vexans*, *Coquillettidia richardii*, *Cx. modestus, Cx. pipiens* complex/*Cx. torrentium* (unspecified to forma level), *Cx. pipiens* f. *pipiens*, *Cx. pipiens f. molestus, Cx. pipiens* f. *pipiens*/f. *molestus* hybrid, *Cx. torrentium*, *Cx. martinii*, *Ochlerotatus geniculatus*, *Ae. cinereus*/*geminus* and *An. plumbeus* (Table 1). Avian *Plasmodium* was not found in the following 14 species: *An. claviger*, *An. hyrcanus*, *An. maculipennis* complex, *Cs. annulata*, *Cx. territans, Oc. cantans*, *Oc. caspius*, *Oc. cataphylla*, *Oc. communis*, *Oc. flavescens*, *Oc. intrudens*, *Oc. japonicus*, *Oc. leucomelas* and *Uranotaenia unguiculata*.

**Table 1:** Total *Plasmodium* prevalence and lineage diversity found during 2013–2015 in mosquitoes sampled in Vienna and Eastern Austria

Species	n individuals	n pools	n *Plasmodium* positive pools	% positive pools	MIR	n Linn1	n SGS1	n SYAT05	n GRW6	n DELURB4	n DONANA03	n unidentified lineages	n mix of lineages
*Ae. cinereus/geminus*	393	32	2	6.25	0.51							2	
*Ae. vexans*	7797	366	2	0.55	0.03	1							1
*Ae./Oc.* sp.	883	74	1	1.35	0.11								1
*An. plumbeus*	35	82	1	1.22	0.28	1 (dominant)							1 (smaller background peaks)
*An.* sp.	410	36	1	2.78	0.24							1	
*Cq. richiardii*	15,846	596	5	0.84	0.03	1		2				2	
*Cx. martinii*	1062	44	1	2.27	0.09								1
*Cx. modestus*	101	27	3	11.11	2.97						1	2	
*Cx. pipiens* s.l.*/torrentium*	13,792	883	136	15.40	0.99	26	22	36	7	1	1	4	39
*Cx.* sp.	410	78	15	19.23	3.66	5	1	5	2				2
*Oc. geniculatus*	47	32	2	6.25	4.26							2	
Others	4652	378	0	0	0								
Total	45,428	2628	169	6.43	0.37	34	23	43	9	1	2	13	45

Minimum infection rate (MIR) is calculated by MIR (%) = n_(PCR positive pools)/n_ _(total analysed mosquitoes)_ × 100 [4]

### Avian *Plasmodium* lineage diversity

Six different genetic lineages of avian *Plasmodium* were found, with the most common *P. vaughani* SYAT05 (n = 43, 25.75% of total infections), *Plasmodium* sp. Linn1 (n = 34, 20.36%) and *P. relictum* SGS1 (n = 23, 13.78%) and *P. elongatum* GRW6 (n = 9, 5.32%) (Table 1). Only three pools (0.11%) were positive for two other lineages, *Plasmodium* sp. DELURB4 and *Plasmodium* sp. DONANA03 (GenBank Accession Numbers: MF347696–MF347701). A total of 45 (26.95%) pools contained more than one haemosporidian lineage, as could be seen on the electropherogram where different peaks superimposed on each other. In addition, one *Cx. pipiens* pool in April 2015 was also positive for one lineage of *Leucocytozoon* sp. TUPHI05 (GenBank Accession Number: MF347702).

### *Plasmodium* prevalence

In 2013, only 11 (12.36%) of a total of 89 mosquito pools were detected positive for avian *Plasmodium*, using a sampling method which provided dried specimens stored at room temperature. Two positive pools for each *Ae. cinereus*/*geminus*, *Cq. richiardii*, *Cx. pipiens* s.l./*torrentium* and *Oc. geniculatus* were detected. Of *Cx. modestus*, three pools were positive for avian *Plasmodium* (Table 2). Of these 11 positives, only three could be sequenced successfully, with one finding of *Plasmodium* sp. DONANA03 in *Cx. modestus* and two in *Cx. pipiens* s.l.*/torrentium*, which were mixes of several lineages with one presenting with *Plasmodium* sp. DONANA03 and the other with *Plasmodium* sp. SYAT38 as dominant lineage.

**Table 2:** Avian *Plasmodium* in different mosquito species in Vienna and Eastern Austria during different sampling events in the years 2013–2015

Mosquito species	2013					2014 Sampling event 1					2014 Sampling event 2					2015				
	n indiv	n pools	n pos. pools	% pos. pools	MIR	n indiv	n pools	n pos. pools	% pos. pools	MIR	n indiv	n Pools	n pos. pools	% pos. pools	MIR	n indiv	n pools	n pos. pools	% pos. pools	MIR
*Ae. cinereus/geminus*	6	5	2	40.00	33.33	6	5				348	16				33	6			
*Ae. vexans*	1718	89				1871	100				4420	152	1	0.66	0.02	1178	62	1	1.61	0.08
*Ae./Oc.*sp.											784	60	1	1.67	0.13	217	27			
*An. plumbeus*	9	6				14	8				150	30				196	42	1	2.38	0.51
*An.* sp.											22	8				388	28	1	3.57	0.26
*Cq. richiardii*	2169	108	2	1.85	0.09	4357	203				1287	68	1	1.47	0.08	8033	217	2	0.92	0.02
*Cx. pipiens* s.l*./torrentium*	2707	151	2	1.32	0.07	1798	116	9	7.76	0.5	2114	325	45	13.85	2.13	7178	291	80	27.49	1.11
*Cx. martinii*											66	11				996	33	1	3.03	0.10
*Cx. modestus*	31	14	3	21.43	9.68	7	6				2	1				61	7			
*Cx.* sp.						4	3	1	33.33	25	131	41	5	12.2	3.82	275	31	9	29.03	3.27
*Oc. geniculatus*	4	4	2	50.00	50.0	17	9				20	13				6	6			
Others	726	63				330	57				1231	105				839	100			
Total	7370	440	11	2.50	0.15	8404	507	10	1.97	0.12	10,575	830	53	6.39	0.5	19,400	850	95	11.18	0.49

In 2014, two different sampling efforts were performed in parallel. In the first, 8404 individuals in 507 pools were collected. Of these, 10 (1.97%) were positive for avian *Plasmodium*. Nine of the positives were found in *Cx. pipiens* s.l./*torrentium*, while the remaining positive was in a pool of unidentified *Culex* mosquitoes (Table 2). Nine of the 10 could be sequenced, presenting with two each of the lineages *Plasmodium* sp. Linn1 and *P. vaughani* SYAT05. The remaining found lineages was one of each *P. relictum* SGS1, *P. elongatum* GRW6 and *Plasmodium* sp. DONANA03.

The second, larger, sampling effort in 2014 collected 10,575 individual mosquitoes in 830 pools; of these, 53 (6.39%) were positive for avian *Plasmodium* (Table 2). Of 2114 individual mosquitoes in 325 pools of *Cx. pipiens* s.l./*torrentium*, genetic identification was performed and it was determined that 91.72% (1939 individuals in 221 pools) belonged to the subspecies *Cx. pipiens* f. *pipiens*, 2.03% (n = 43, 26 pools) belonged to *Cx. pipiens* f. *molestus*, 3.6% (n = 76, 45 pools) were hybrids of the former, and 2.65% (n = 56, 33 pools) were of the species *Cx. torrentium*. Of the 325 pools in this group, 13.85% (n = 45) were positive for *Plasmodium* (Table 3). The majority of *Plasmodium* positives were found in pools of *Cx. pipiens* f. *pipiens*, with 16.74% (n = 37) positive. Only 3.85% (n = 1) of *Cx. pipiens* f. *molestus*, 8.89% (n = 4) of the *Cx. pipiens* f. *pipiens/*f. *molestus* hybrids and 9.09% (n = 3) of *Cx. torrentium* were positive (Table 2). Only two other identified species of mosquito were found positive for avian *Plasmodium* during this sampling effort in 2014, *Ae. vexans* and *Cq. richardii*. One positive pool of an unidentified lineage mix (0.66%) was found in *Ae. vexans* and one pool (1.47%) was positive for *P. vaughani* SYAT05 in *Cq. richardii*.

**Table 3:** Avian *Plasmodium* prevalence and lineage diversity found in 2014 in mosquitoes of *Culex pipiens* s.l. and *Culex torrentium*, sampled in Vienna and Eastern Austria

Mosquito species	n individuals	n pools	*Plasmodium* positive pools	% positive pools	MIR	n Linn1	n SGS1	n SYAT05	n GRW6	n mix of lineages
*Cx. pipiens* f. *pipiens*	1939	221	37	16.74	1.91	6	7	11	2	11
*Cx. pipiens* f. *molestus*	43	26	1	3.85	2.33			1		
*Cx. pipiens* f. *pipiens/*f. *molestus* hybrid	76	45	4	8.89	5.26			3		1
*Cx. torrentium*	56	33	3	9.09	5.36			3		
Total	2114	325	45	13.85	2.13	6	7	18	2	12

Minimum infection rate (MIR) is calculated by MIR (%) = number of PCR positive pools/total number of analysed mosquitoes × 100 [4]

In 2015, 19,400 mosquito individuals, divided into 850 pools, were collected. A total of 95 pools (11.18%) of 5 identified species and one pool of unidentified mosquitoes of the genus *Anopheles* and 9 pools of unidentified *Culex* mosquitoes were positive for avian *Plasmodium* (Table 3). In one pool of the *Cx. pipiens* complex, *Leucocytozoon* sp. TUPHI05 was found. The majority of positives (n = 80) were found in *Cx. pipiens* s.l./*torrentium* with 27.49%. This group also contained the highest *Plasmodium* diversity, with 5 different lineages found (Linn1, SGS1, GRW11, GRW6, DELURB4). Two pools (0.92%) of *Cq. richiardii* were also positive, as well as one pool each of *Ae. vexans* (1.61%), *An. plumbeus* (2.38%) and *Cx. martinii* (3.03%).

### Minimum infection rate (MIR)

**Fig. 2:**
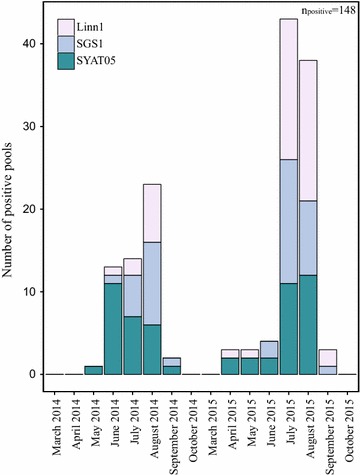
Avian *Plasmodium* numbers in Eastern Austrian mosquito pools during the sampled months for the second sampling effort in 2014 and 2015. Shown is the total number of *Plasmodium* infections for each month as well as the numbers of the most common lineages Linn1, SGS1 and SYAT05

**Fig. 3:**
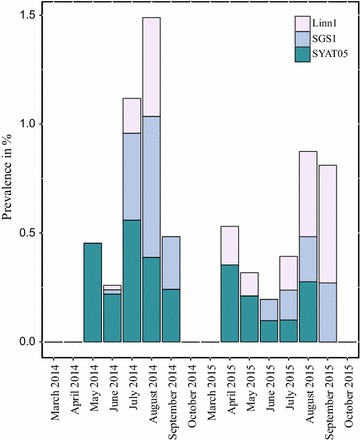
Minimum infection rate (MIR) for avian *Plasmodium* in Eastern Austrian mosquitoes during the sampled months for the second sampling effort in 2014 and 2015. Shown is the total MIR (%) for all *Plasmodium* infections as well as the MIR for the most common lineages Linn1, SGS1 and SYAT05

The MIR varied between different mosquito species and between the years and different sampling events (Tables 2 and 3, Figs. 2 and 3). The average total MIR during all years and sampling events was 0.37%, with the highest averages for *Oc. geniculatus* (4.26%), *Cx. modestus* (2.97%) and *Cx. pipiens* s.l./*torrentium* (0.99%) (Table 1). The highest overall MIR was found in *Oc. geniculatus* (50%) and *Ae. cinereus/geminus* (33.33%) in 2013 (Table 2). In 2014, where mosquitoes of the *Cx. pipiens* complex and *Cx. torrentium* were genetically identified, it was also possible to determine the MIR in the different biotypes comprising this complex (Table 3). Here, *Cx. torrentium* presented with the highest MIR (5.36%), followed by *Cx. pipiens* f. *pipiens*/f. *molestus* hybrids (5.26%), *Cx. pipiens f. molestus* (2.33%) and finally *Cx. pipiens* f. *pipiens* (1.91%).

### Monthly changes and differences between 2014 and 2015

When comparing avian *Plasmodium* infections in different months in 2014 and 2015, only taking the second sampling event for 2014 into account (Table 2), total number and prevalence was highest in summer (August) in both years (Fig. 2). The same is true for the MIR in 2014, while the MIR in 2015 was highest in September, which was also the highest observed MIR of all sampled months of both years (Fig. 3). In 2014, a steep increase in the total number of infections appeared in June, while in 2015, this increase appeared 1 month later in July. Also, in 2014, the total number of infections with lineage *P. vaughani* SYAT05 was highest in June, where it dominated the lineage assembly, while later in the year, in August, *P. relictum* SGS1 became dominant (Fig. 2). In 2015, this could not be observed and the lineage *Plasmodium* sp. Linn1 dominated both July and August. When comparing the prevalence and MIR of different lineages over the different months (Figs. 2 and 3), *P. vaughani* SYAT05 was also predominant in the early sampling period (May to July) in 2014, while in August, *P. relictum* SGS1 dominated. Similarly, in 2015, *P. vaughani* SYAT05 prevalence was predominant in the beginning of the sampling period (April); but as was also seen in total number of infections in later months (July, August, September) of 2015, *Plasmodium* sp. Linn1 dominated.

## Discussion

### *Plasmodium* lineage diversity

This is the first large-scale study of avian *Plasmodium* parasites in Austrian mosquitoes. The majority of avian *Plasmodium* found belonged to four very commonly detected lineages, *P. relictum* SGS1, *P. elongatum* GRW6, *P. vaughani* SYAT05 and *Plasmodium* sp. Linn1. These have also been reported in several previous studies on mosquito vectors in Europe. SYAT05 was found in *Cx. pipiens* (L.) in the Czech Republic [30] and Switzerland [12], in *Culex modestus* in Spain [17] and in *Cx. pipiens* (L.) and *Culex theileri* in Portugal [5]. All three were found, among other lineages, in *Cx. pipiens* (L.) in France [21] and Switzerland [3]. A range of other avian malaria lineages has been found in *Cx. pipiens* in Italy [19], Switzerland [3], France [21], and Turkey [18].

Only a total of three of the positive pools contained the European lineages *Plasmodium* sp. DELURB4 and DONANA03. Since whole mosquitoes were used in pools, it was not possible to assert competent vector status on the sampled mosquito species. More than a quarter of all positive mosquito pools examined in this study carried a mix of either two or more of the most common *Plasmodium* lineages. Detecting mixes in this study is a by-product of examining pools of mosquitoes instead of looking at individuals, although the presence of several lineages, visible as double peaks on the chromatogram, has been found in other studies examining single mosquitoes [21]. Since mixed infections are also very common in birds [31, 32], mixed infections in individual mosquitoes should also be expected.

It can be assumed that during our study, the true diversity of avian *Plasmodium* in Austrian mosquitoes was greatly underestimated, a direct result of examining pooled samples where the presence of more than one parasite lineage is to be expected. Although PCR has greatly improved the detection of haemosporidian infections, it is still far from perfect. When examining infected bird blood, Jarvi et al. [33] noted that PCR diagnostics underestimated the prevalence of avian malaria in experimentally infected passerines. In addition, the acquired sequence may be of a light infection that amplifies better with the PCR protocol used, but is not identical to the parasite seen under the microscope [34]. In addition, PCR sometimes fails to amplify DNA of a clearly visible and even predominant parasite in blood samples [35]. Conventional PCR assays also underestimate mixed infections, which are common in the wild, because they might preferably amplify the DNA of one parasite over another [36].

### Avian *Plasmodium* prevalence, minimum infection rate and seasonal changes

The overall prevalence for avian malaria found in the examined mosquito pools was 6.43%. When looking at prevalence in the mosquito species, these findings are comparable with others worldwide. Two studies in Switzerland looking at *Cx. pipiens* for example found an overall prevalence of 6.6% (n = 394) in 2006/2007 [12] and 13.1–20.3%, depending on the season in 2010/2011 [3]. In France, looking at the same mosquito species, prevalences ranging from 0% (February) to 15.8% (October) were observed [21]. A study in the USA, looking at different mosquito species, found 10% of 61 pools positive [37]. In studies in Japan, prevalence ranged from 14.3 to 23.9% depending on area and examined mosquito species [38–40]. Still, the result presented here is only an estimate of prevalence. When examining pooled samples, there is a chance that true prevalence is overlooked, as demonstrated in a study on mosquitoes in Vanuatu and New Caledonia [41], where total prevalence was twice when examining single individuals compared to examining pooled samples.

The MIR for different species of mosquitoes is generally very variable, and can range for example from 3.08% [39] to 0.52% [38] and 0.03% [5] in *Cx. pipiens.* In other species, such as *Culex bitaeniorhynchus*, MIR can reach as high as 6% [42] or as high as 13% [37]. The MIR reported in here was therefore consistent with findings in other mosquito species worldwide [37, 42, 43]. The high MIR found in *Ae. cinereus*/*geminus* and *Oc. geniculatus* during this study in 2013 was most likely due to the low sample size of these species collected in 2013, which skewed the result.

In general, the highest number of avian *Plasmodium* positives was found in late summer (August). In the northern hemisphere, most transmission of the parasites between birds occurs during the breeding season, when vector populations are increasing with the onset of warm weather. During that time, adult birds with chronic relapsing infections are available as sources of infection, and non-immune juvenile birds are hatching and leaving the nest [44]. In a study on *Cx. pipiens* (L.) in Switzerland, female mosquitoes caught in summer were more likely to be infected than females caught in spring [3]. Similar results were found in a study in Spain, where parasite prevalence in vectors was highest in autumn and lowest in spring [17]. In 2014 and 2015, differences were also found in dominance of several *Plasmodium* lineages during the examined months. In 2014, the total number of infections with lineage *P. vaughani* SYAT05 was highest in June, where it dominated the lineage assembly, while later in the year, in August, *P. relictum* SGS1 became dominant. In 2015, this could not be observed and the lineage *Plasmodium* sp. Linn1 dominated both July and August. This is comparable to findings of Labulin et al. [3], who showed that the dominance of different *Plasmodium* species within the studied vector population varied during the seasons. While the total prevalence increased, infections with *P. vaughani* SYAT05 decreased from spring to summer to be replaced by different *P. relictum* lineages SGS1, GRW11 and PADOM02. They argued that this might be due to seasonal changes in host-feeding preferences, the development of immunity in the vector against different lineages or different lineages developing under different environmental conditions.

The variations found in lineage distribution and prevalence comparing different years may be explained with an annual variation in vector abundance and activity. The feeding activity of dipteran vectors depends on weather conditions and can be affected by temperature and wind. Another explanation for the annual changes might be the winter mortality of the vertebrate hosts. Blood parasites may cause pathology in the host [9, 45], and during winter, uninfected birds may survive better than infected ones [46].

In general, samples collected with method 1 in 2013 and 2014 and stored as dry specimens yielded far less positives than samples collected with method 2 and it is likely that the DNA quality was compromised by the storage method, as shown in a previous study by Werblow et al. [47].

### Avian *Plasmodium* in mosquitoes of the *Culex pipiens* complex s.l./*Culex torrentium*

The second-most common mosquitoes caught in this study were taxa belonging to the morphologically indistinguishable *Cx. pipiens* complex and *Cx. torrentium*. During a previous study, mosquitoes of this species complex sampled in 2014 were identified genetically [16] and this provided an opportunity to determine avian *Plasmodium* diversity and prevalence in these dipterans. During sampling, the most common mosquito of this species group caught was *Cx. pipiens* f. *pipiens* and subsequently, the largest total number of avian *Plasmodium* parasites as well as the largest proportion of positive pools was found in this species. However, when comparing the MIR of the different species and the hybrids in the species complex, differences are evident. *Culex torrentium* showed the highest MIR, followed by the *Cx. pipiens* f. *pipiens*/f. *molestus* hybrids, while the MIR for *Cx. pipiens* was lowest. It is unclear if these differences could be explained by the much lower sample size of *Cx. pipiens* f. *molestus*, *Cx. torrentium* and hybrids or if these mosquitoes in general bite birds more frequently and therefore have a higher chance of acquiring avian *Plasmodium*.

In general, mosquitoes of this species complex provided the majority of avian *Plasmodium* detected during this study. This finding is not surprising, since the genus *Culex* provides the most successful vectors for these parasites worldwide [10, 12]. In addition, mosquitoes of the *Cx. pipiens* complex and their hybrids frequently bite birds [6] and have therefore the opportunity to acquire avian *Plasmodium* with a blood meal.

One lineage of *Leucocytozoon* sp. TUPHI05 in one pool of undetermined mosquitoes of the species complex was also detected. Members of the genus *Leucocytozoon* are haemosporidian parasites, which are closely related to *Plasmodium* and also infect birds, although they are transmitted by black flies of the family Simuliidae [1]. It is not unusual to find haemosporidian parasites in vectors that usually do not transmit them, especially when whole insects are examined and vector competence cannot be asserted [6].

## Conclusions

To gain further insight into avian *Plasmodium* prevalence, distribution and diversity in mosquitoes in Austria, further research is required. New molecular detection methods are needed to reliably and simultaneously detect different lineages in a single sample. For a better understanding of parasite transmission and vector competence, experimental infections as well as the examination of mosquito thoraxes and salivary glands for avian *Plasmodium* are needed. Examining individual mosquitoes would be ideal for detecting the real diversity of *Plasmodium* parasites [10, 21], but this approach is costly and many research groups use pooled samples [40, 48, 49]. In the case of blood-fed mosquitoes, individuals have to be processed individually to determine the bitten bird host [40, 50]. It is high time to carry out such studies now, as data collected in the near future will provide the basis for the evaluation of long-term changes to the system bird-vector-parasite due to human impacts (changes and/or destruction of bird habitat) and changes in bird and vector distributions due to predicted climate change.

## References

[1] Valkiunas G (2005). Avian malaria parasites and other Haemosporidia.

[2] Clark NJ, Clegg SM, Lima MR (2014). A review of global diversity in avian haemosporidians (*Plasmodium* and *Haemoproteus*: Haemosporida): new insights from molecular data. Int J Parasitol.

[3] Lalubin F, Deledevant A, Glaizot O, Christe P (2013). Temporal changes in mosquito abundance (*Culex pipiens*), avian malaria prevalence and lineage composition. Parasit Vectors..

[4] Marzal A, Bensch S, Reviriego M, Balbontin J, de Lope F (2008). Effects of malaria double infection in birds: one plus one is not two. J Evol Biol.

[5] Ventim R, Ramos JA, Osorio H, Lopes RJ, Perez-Tris J, Mendes L (2012). Avian malaria infections in western European mosquitoes. Parasitol Res.

[6] la Puente Martínez-de, Ferraguti M, Ruiz S, Roiz D, Soriguer RC, Figuerola J (2016). *Culex pipiens* forms and urbanization: effects on blood feeding sources and transmission of avian *Plasmodium*. Malar J..

[7] Hellgren O, Perez-Tris J, Bensch S (2009). A jack-of-all-trades and still a master of some: prevalence and host range in avian malaria and related blood parasites. Ecology.

[8] Hellgren O, Atkinson CT, Bensch S, Albayrak T, Dimitrov D, Ewen JG (2015). Global phylogeography of the avian malaria pathogen *Plasmodium relictum* based on MSP1 allelic diversity. Ecography.

[9] Dinhopl N, Nedorost N, Mostegl MM, Weissenbacher-Lang C, Weissenbock H (2015). In situ hybridization and sequence analysis reveal an association of *Plasmodium* spp. with mortalities in wild passerine birds in Austria. Parasitol Res.

[10] Kimura M, Darbro JM, Harrington LC (2010). Avian malaria parasites share congeneric mosquito vectors. J Parasitol.

[11] LaPointe DA, Goff ML, Atkinson CT (2005). Comparative susceptibility of introduced forest dwelling mosquitoes in Hawai’i to avian malaria, Plasmodium relictum. J Parasitol.

[12] Glaizot O, Fumagalli L, Iritano K, Lalubin F, Van Rooyen J, Christe P (2012). High prevalence and lineage diversity of avian malaria in wild populations of great tits (*Parus major*) and mosquitoes (*Culex pipiens*). PLoS ONE.

[13] Bensch S, Hellgren O, Perez-Tris J (2009). MalAvi. A public database of malaria parasites and related haemosporidians in avian hosts based on mitochondrial cytochrome b lineages. Mol Ecol Res..

[14] Zittra C, Joachim A, Fuehrer H-P (2015). Mosquitoes and *Dirofilaria* in Austria-a review of the current situation of neobiotic Culicidae and Dirofilariae. Tierärztliche Umschau..

[15] Zittra C, Obwaller AG, Wimmer V, Berer D, Eigner B, Fuehrer HP (2017). First record of *Orthopodomya pulcripalpis* (Rondani, 1872) (Diptera: Culicidae) in Austria. Parasitol Res.

[16] Becker N, Petrić D, Boase C, Lane J, Zgomba M, Dahl C, Kaiser A (2003). Mosquitoes and their control.

[17] Ferraguti M, Martinez-de la Puente J, Munoz J, Roiz D, Ruiz S, Soriguer R, Figuerola J (2013). Avian *Plasmodium* in *Culex* and *Ochlerotatus* mosquitoes from Southern Spain: effects of season and host-feeding source on parasite dynamics. PLoS ONE.

[18] Inci A, Yildirim A, Njabo K, Duzlu O, Biskin Z, Ciloglu A (2012). Detection and molecular characterization of avian *Plasmodium* from mosquitoes in central Turkey. Vet Parasitol.

[19] Martinez-de la Puente J, Munoz J, Capelli G, Montarsi F, Soriguer R, Arnoldi D (2015). Avian malaria parasites in the last supper: identifying encounters between parasites and the invasive Asian mosquito tiger and native mosquito species in Italy. Malar J..

[20] Santiago-Alarcon D, Palinauskas V, Schaefer HM (2012). Diptera vectors of avian Haemosporidian parasites: untangling parasite life cycles and their taxonomy. Biol Rev.

[21] Zele F, Vezilier J, L’Ambert G, Nicot A, Gandon S, Rivero A (2014). Dynamics of prevalence and diversity of avian malaria infections in wild *Culex pipiens* mosquitoes: the effects of Wolbachia, filarial nematodes and insecticide resistance. Parasit Vectors..

[22] Ziegyte R, Bernotiene R, Bukauskaite D, Palinauskas V, Iezhova T, Valkiunas G (2014). Complete sporogony of *Plasmodium relictum* (lineages pSGS1 and pGRW11) in mosquito *Culex pipiens pipiens* form *molestus*, with implications to avian malaria epidemiology. J Parasitol.

[23] Valkiunas G, Ziegyte R, Palinauskas V, Bernotiene R, Bukauskaite D, Ilgunas M (2015). Complete sporogony of *Plasmodium relictum* (lineage pGRW4) in mosquitoes *Culex pipiens pipiens*, with implications on avian malaria epidemiology. Parasitol Res.

[24] Kazlauskiene R, Bernotiene R, Palinauskas V, Iezhova TA, Valkiunas G (2013). *Plasmodium relictum* (lineages pSGS1 and pGRW11): complete synchronous sporogony in mosquitoes *Culex pipiens pipiens*. Exp Parasitol.

[25] Zittra C, Flechl E, Kothmayer M, Vitecek S, Rossiter H, Zechmeister T (2016). Ecological characterization and molecular differentiation of *Culex pipiens* complex taxa and *Culex torrentium* in eastern Austria. Parasit Vectors..

[26] Hellgren O, Waldenstrom J, Bensch S (2004). A new PCR assay for simultaneous studies of *Leucocytozoon*, *Plasmodium*, and *Haemoproteus* from avian blood. J Parasitol.

[27] Kearse M, Moir R, Wilson A, Stones-Havas S, Cheung M, Sturrock S, Buxton S (2012). Geneious Basic: an integrated and extendable desktop software platform for the organization and analysis of sequence data. Bioinformatics.

[28] Benson DA, Karsch-Mizrachi I, Lipman DJ, Ostell J, Sayers EW (2011). GenBank. Nucleic Acids Res.

[29] White BJ, Andrew DR, Mans NZ, Ohajuruka OA, Garvin MC (2006). West Nile Virus in mosquitoes of northern Ohio, 2003. Am J Trop Med Hyg.

[30] Synek P, Munclinger P, Albrecht T, Votypka J (2013). Avian haemosporidians in haematophagous insects in the Czech Republic. Parasitol Res.

[31] Jarvi SI, Farias ME, Atkinson CT (2008). Genetic characterization of Hawaiian isolates *of Plasmodium relictum* reveals mixed-genotype infections. Biol Direct..

[32] Van Rooyen J, Lalubin F, Glaizot O, Christe P (2013). Avian haemosporidian persistence and co-infection in great tits at the individual level. Malar J..

[33] Jarvi SI, Schultz JJ, Atkinson CT (2002). PCR diagnostics underestimate the prevalence of avian malaria (*Plasmodium relictum*) in experimentally-infected passerines. J Parasitol.

[34] Valkiūnas G, Zehtindjiev P, Hellgren O, Ilieva M, Iezhova TA, Bensch S (2007). Linkage between mitochondrial cytochrome b lineages and morphospecies of two avian malaria parasites, with a description of *Plasmodium* (*Novyella*) *ashfordi* sp. nov. Parasitol Res.

[35] Zehtindjiev P, Krizanauskiene A, Bensch S, Palinauskas V, Asghar M, Dimitrov D (2012). A new morphologically distinct avian malaria parasite that fails detection by established polymerase chain reaction-based protocols for amplification of the cytochrome b gene. J Parasitol.

[36] Valkiunas G, Bensch S, Iezhova TA, Krizanauskiene A, Hellgren O, Bolshakov CV (2006). Nested cytochrome B polymerase chain reaction diagnostics underestimate mixed infections of avian blood haemosporidian parasites: microscopy is still essential. J Parasitol.

[37] Fryxell RTT, Lewis TT, Peace H, Hendricks BBM, Paulsen D (2014). Identification OF avian malaria (*Plasmodium* sp.) and canine heartworm (*Dirofilaria immitis*) in the mosquitoes of Tennessee. J Parasitol.

[38] Ejiri H, Sato Y, Sawai R, Sasaki E, Matsumoto R, Ueda M (2009). Prevalence of avian malaria parasite in mosquitoes collected at a zoological garden in Japan. Parasitol Res.

[39] Kim KS, Tsuda Y (2010). Seasonal changes in the feeding pattern of *Culex pipiens pallens* govern the transmission dynamics of multiple lineages of avian malaria parasites in Japanese wild bird community. Mol Ecol.

[40] Kim KS, Tsuda Y, Yamada A (2009). Bloodmeal identification and detection of avian malaria parasite from mosquitoes (Diptera: Culicidae) inhabiting coastal areas of Tokyo Bay, Japan. J Med Entomol.

[41] Ishtiaq F, Guillaumot L, Clegg S, Phillimore A, Black R, Owens I, Mundy N (2008). Avian haematozoan parasites and their associations with mosquitoes across Southwest Pacific Islands. Mol Ecol.

[42] Kim KS, Tsuda Y (2012). Avian *Plasmodium* lineages found in spot surveys of mosquitoes from 2007 to 2010 at Sakata wetland, Japan: do dominant lineages persist for multiple years?. Mol Ecol.

[43] Ejiri H, Sato Y, Kim K-S, Hara T, Tsuda Y, Imura T (2011). Entomological study on transmission of avian malaria parasites in a zoological garden in Japan: bloodmeal identification and detection of avian malaria parasite DNA from blood-fed mosquitoes. J Med Entomol.

[44] Atkinson CT, van Riper C, Loye JE, Zuk M (1991). Pathogenicity and epizootiology of avian haematozoa: *Plasmodium*, *Leucocytozoon*, and *Haemoproteus*. Bird-parasite interactions: ecology, evolution, and behavior.

[45] Ilgūnas M, Bukauskaitė D, Palinauskas V, Iezhova TA, Dinhopl N, Nedorost N (2016). Mortality and pathology in birds due to *Plasmodium* (*Giovannolaia*) *homocircumflexum* infection, with emphasis on the exoerythrocytic development of avian malaria parasites. Malar J..

[46] Allander K, Bennett GF (1994). Prevalence and intensity of haematozoan infection in a population of great tits *Parus major* from Gotland, Sweden. J Avian Biol.

[47] Werblow A, Flechl E, Klimpel S, Zittra C, Lebl K, Kieser K (2016). Direct PCR of indigenous and invasive mosquito species: a time-and cost-effective technique of mosquito barcoding. Med Vet Entomol.

[48] Njabo KY, Cornel AJ, Bonneaud C, Toffelmier E, Sehgal RNM, Valkiūnas G (2011). Nonspecific patterns of vector, host and avian malaria parasite associations in a central African rainforest. Mol Ecol.

[49] Njabo KY, Cornel AJ, Sehgal RNM, Loiseau C, Buermann W, Harrigan RJ (2009). *Coquillettidia* (Culicidae, Diptera) mosquitoes are natural vectors of avian malaria in Africa. Malar J..

[50] Hamer GL, Kitron UD, Goldberg TL, Brawn JD, Loss SR, Ruiz MO (2009). Host selection by *Culex pipiens* mosquitoes and West Nile virus amplification. Am J Trop Med Hyg.

